# Understanding the knowledge, attitudes, and practices of stakeholders in reporting African swine fever cases in Abuyog, Leyte, Philippines

**DOI:** 10.5455/javar.2025.l927

**Published:** 2025-06-02

**Authors:** Valine A. Cabodil, Harvie P. Portugaliza

**Affiliations:** Department of Veterinary Clinical Sciences, Faculty of Veterinary Medicine, Visayas State University, Baybay, Philippines

**Keywords:** African swine fever, knowledge, attitudes, practices, pig farming.

## Abstract

**Objective:**

This study aimed to assess the knowledge, attitudes, and practices (KAP) of key stake-holders regarding African swine fever (ASF) and its reporting in Abuyog, Leyte, Philippines. It also aimed to identify sociodemographic factors associated with KAP levels.

**Materials and Methods:**

A cross-sectional survey was performed on 392 respondents, including pig farmers (*n* = 333), butchers (*n* = 38), live pig/meat sellers (*n* = 11), and Local Government Unit personnel (*n* = 10) between November 2023 and February 2024. KAP scores were calculated and categorized into “poor” and “good” using a median cutoff. Logistic regression analyses were conducted to investigate the association between sociodemographic variables and KAP levels.

**Results:**

Most participants showed poor knowledge of ASF causative agents, transmission, and clinical signs (83.93%) and disease recognition (60.20%), but many have good knowledge of ASF reporting protocols (70.92%). Attending ASF seminars/training was associated with improved basic ASF knowledge, disease recognition, and case reporting. Basic knowledge of ASF could enhance disease recognition. Disease recognition could then enhance ASF case reporting. Younger stakeholders showed better knowledge of basic ASF concepts. Pig farmers exhibited poor knowledge of disease recognition. Most participants showed good attitudes toward ASF reporting (97.7%), which was associated with overall knowledge of ASF. Most participants showed good practices in the early steps of case reporting (85.20%), relatively balanced on reporting protocol (49.23%), and relatively poor knowledge-seeking behavior (45.41%). Pig farmers were less likely to report than other stakeholders. Good overall knowledge translates into good practices. Overall practices are influenced by the primary source of income.

**Conclusion:**

The findings reveal a notable gap in knowledge concerning ASF among participants, highlighting an essential need for enhanced educational initiatives. Strengthening basic ASF knowledge is vital, as it positively impacts disease recognition and, in turn, case reporting. Although there is a generally positive attitude toward ASF reporting, the lack of knowledge-seeking behavior and the variability in reporting practices based on income sources suggest a need for tailored educational programs.

## Introduction

African swine fever (ASF) is a highly contagious disease of pigs caused by the ASF virus (ASFV), an icosahedral DNA virus from the family Asfarviridae. Although ASF is not zoonotic, it leads to massive mortality in pigs, causing significant economic losses. Developing vaccines has posed significant challenges over the past few decades, but recent advancements in gene-deleted live attenuated vaccines have demonstrated notable effectiveness against ASF. One such vaccine, ASFV-G-∆I177L, was developed in Vietnam and is now available on the market, although it is not widely accessible in many countries [1,2]. In the Philippines, the Department of Agriculture (DA) reported the first ASF outbreak in July 2019 in Rizal. By September 2019, the ASFV had spread and affected several provinces and barangays in Luzon. Despite early warning contingency plans and prevention strategies imposed by other regions, the disease reached Mindanao in January 2020 [3]. By January 2021, the DA confirmed the detection of ASF in Barangay Can-aporong, Abuyog, Leyte, the first case reported in the Visayas Region [4].

Control measures against ASF are hampered by the complex epidemiology of the disease, the difficulty of applying strict biosecurity measures, the lack of adequate and safe vaccines, no specific treatments, and high-risk practices that remain in small and non-commercial farming sectors [5], thus requiring a transdisciplinary approach [6,7]. An essential factor in managing and preventing this disease is the involvement of stakeholders in the pig industry, such as farm owners, in detecting and reporting cases. Early detection of the virus’s entry limits the extent of a possible outbreak. However, timely and accurate reporting of primary ASF cases depends on swine farmers’ familiarity with their clinical signs and motivations for reporting the disease [8]. Despite government efforts, many ASF cases remain unreported. Farmers in the Philippines, especially smallholders, chose not to report suspicious cases or deaths on the farm because they were concerned about losses not covered by the government [9], which may lead to the slaughtering of potentially infected pigs for consumption and sale [10]. Of note, the Philippine government has policies to aid farmers affected by ASF through the Philippine Crop Insurance Corporation and an indemnification program for pigs that are depopulated as part of the measures to control ASF outbreaks.

The primary aim of this study is to evaluate the knowledge, attitudes, and practices (KAP) of key stakeholders in the pig production system in Abuyog, Leyte, regarding ASF and its case reporting. Additionally, the study aims to identify sociodemographic factors that may influence KAP levels. The findings from this study are anticipated to provide insights for addressing barriers to ASF reporting and enhancing the efficacy of early warning contingency plans for neighboring administrative areas in controlling ASF.

## Materials and Methods

### Ethical approval

This study received approval from the VSU’s Ethics Review Committee (ERC) (REC code 2023-22-CVM). Participants were given informed consent before the interview. They were made aware that answering the survey questionnaire was voluntary and that no judicial or social consequences would be applied to those who did not participate. All collected data were treated according to the Philippine R.A. 10173, or the Data Privacy Act of 2012.

### General description of the study area

**Figure 1.**
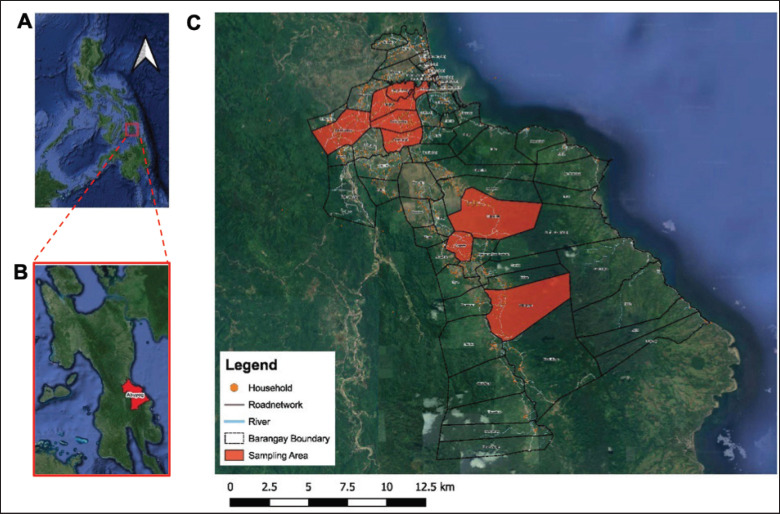
Map of the study sites. (A) The Philippines. (B) Leyte Island with red highlight on the municipality of Abuyog in the Leyte Province. (C) The map of the municipality of Abuyog, with red highlights on barangay study sites. Black lines represent the boundaries of each barangay. This map was created using QGIS (qgis.org/en/site/).

The data were gathered from the different respondents of the municipality of Abuyog, Leyte (10° 44’ 7” North, 125° 1’ 17” East). Facing Leyte Gulf out into the Philippine Sea, Abuyog is the largest town on the island of Leyte in terms of land area (68,825 hectares). It is bordered by Javier to the north, by Mahaplag and Baybay City to the south by Silago in Southern Leyte. The municipality of Abuyog, Leyte (Fig. 1), comprises 63 barangays with a population of 61,216 and a household number of 13,294 [11]. The municipality has recorded 1,582 swine farmers, with 7,146 pigs as of June 22, 2021 (data derived from the DA-Local Government Unit of Abuyog, Leyte). All farms enrolled in this study are considered smallholder farms (1–10 sow level or 1–100 heads) based on the categorization of the Philippine Statistics Authority [12], with the average number of one sow and seven heads of pigs.

### Study and sampling design

A cross-sectional survey was conducted to assess KAP levels of selected stakeholders. The sample size was calculated using Cochran’s sample size formula at a 95% confidence level and 5% margin of error [13]. At least 313 respondents were targeted for the survey based on the data presented by the Abuyog, Leyte Municipal Agriculture Office, which documented 1,582 backyard farmers, 18 meat sellers, 23 Local Government Unit (LGU) personnel, and 46 butchers. Proportional allocation was performed to represent each stakeholder [14]. In the survey, 392 respondents were interviewed: 333 farmers, 38 butchers, 11 meat sellers, and 10 LGU personnel.

### Data collection using a KAP questionnaire

A face-to-face survey in English and translated into the local dialect of Waray-Waray was arranged for participants to provide their responses (Supplementary Information 1, 2). Each survey comprised an informative section detailing the study’s nature and a consent form clarifying the confidentiality of participants’ identities and granting authorization to use and analyze provided information. Additionally, a section was included to collect sociodemographic data, such as age, sex, marital status, educational attainment, role in the pork and swine industry, primary source of income, estimated income, and years of experience in pig raising or selling. The face-to-face interviews adhered to the minimum health requirements outlined by the Inter-Agency Task Force.

The KAP questionnaire (Supplementary Information 2) was divided into three sections, adopting the questions from Wheless et al. [9], Vergne et al. [15], and Chenais et al. [16]. The first part comprised the knowledge section, inquiring about respondents’ understanding of the disease and the protocols for reporting suspected cases to authorities. The second part encompassed the attitudes section, probing respondents’ perspectives on the early reporting of suspected ASF cases and their readiness to adhere to suitable protocols for disease prevention. The third part comprised the practices section, exploring respondents’ actions concerning reporting suspected cases, any impacts the disease may have had on their businesses, and any obstacles hindering them from reporting suspected cases.

The pilot testing of the KAP questionnaire conducted in Macarthur, Leyte, Philippines, involved at least 30 respondents to evaluate potential ambiguities in the questions and their efficacy in eliciting the intended information. Subsequently, a Cronbach’s *α* test was employed to assess the internal consistency of the survey questionnaire. The questionnaire demonstrated a Cronbach’s α value of 0.7. A Cronbach’s α value between 0.7 and 0.8 signified adequate reliability [17].

### Data management and analysis

The data from the questionnaires were entered into Microsoft Excel 2013 software for data cleaning and analyzed using JASP version 0.19.0 (https://jasp-stats.org/). Sociodemographic characteristics were examined using descriptive statistics. Continuous variables were described as mean and median, while categorical variables were presented as frequencies and percentages with 95% confidence interval (CI).

The details of the KAP scoring system are presented in Supplementary Information 3. Here, the K score was divided into subcomponents: (1) causative agent, transmission, and clinical signs (CTC); (2) disease recognition; and (3) disease reporting. The overall K score was also calculated by combining these components. The A score was categorized as (1) reporting and (2) consuming and selling, aside from the overall score. The P score was categorized into three subcomponents: (1) early steps in disease reporting, (2) protocol in disease reporting, and (3) attendance at ASF-related seminars and training. The overall P score was also calculated. For scoring of individual responses, answer keys were made based on published data from the World Organization for Animal Health [18] and the Philippine government’s protocols related to ASF (DA Administrative Order No. 7, Series of 2021). The responses for the knowledge section were scored as follows: a “1” for correct answers, “0.5” for partially correct answers, and “0” for incorrect responses. The attitudes section utilized a 5-point Likert scale, allowing respondents to choose from five categories: “strongly agree” scored “5,” “agree” scored “4,” “neutral” scored “3,” “disagree” scored “2,” and “strongly disagree” scored “1.” When appropriate, reverse coding was applied to selected items in the Attitudes section. In the Practices section, responses were scored with “1” for correct practices, “0.5” for partially correct practices, and “0” for incorrect practices. The KAP levels for each respondent were presented as percentages relative to the perfect score based on the standard answers. A classification of poor (< 50%) and good (> 50%) was used for K, A, and P.

To identify factors that may explain variations of KAP levels, a binary logistic regression analysis (LRA) was performed to analyze the association between sociodemographic variables (i.e., independent variables) and each categorized score for K, A, and P (i.e., dependent variables categorized as a poor score if < 50% [coded as 0] and a good score if > 50% [coded as 1]). A univariate LRA was first performed to determine the unadjusted odds ratio (OR) and *p*-value of each independent variable. Next, the independent variables with *p* < 0.20 in the univariate analysis were included in the multivariable LRA. Separate multivariable regression models were created for each component of KAP, using stepwise regression following a forward elimination approach. Independent variables with the least significant *p*-values were removed at each stage of the multivariable regression until a model with significant variables of *p* < 0.05 was obtained, of which the adjusted OR (AOR) with 95% CI, *p*-values, and Cox and Snell *R^2^* were presented. Age and sex were included in all multivariable logistic regression models as forced variables. In addition, the link between knowledge scores and attitudes/practices scores was tested. The knowledge score was included in the multivariable logistic regression models for attitudes and practices.

## Results

### Respondent’s characteristics

The stakeholders’ ages varied between 23 and 86 years, with an average age of 47.45 years (SD = 12.39) and a median of 48.00. The sex distribution was fairly even between males (45.41%) and females (54.59%). Most stakeholders were married, accounting for 72.96% of the respondents. High school was the highest educational attainment of most participants, comprising over half of the participants (51.66%), followed by elementary education (31.20%), college (13.55%), and vocational (3.58%). Agribusiness was the primary source of income for 50.26% of the participants, and on average, they had 12.78 years (SD = 8.37) of experience in the swine sector. The average reported monthly income stood at 7,824.00 Php (SD = 7563) (see also Supplementary Information 4).

### The ASF knowledge score

This study evaluated the K scores based on (1) knowledge regarding the ASF CTC; (2) knowledge of ASF clinical presentation; and (3) knowledge of ASF reporting protocols outlined by the Philippine Bureau of Animal Industry (BAI).

**Figure 2.**
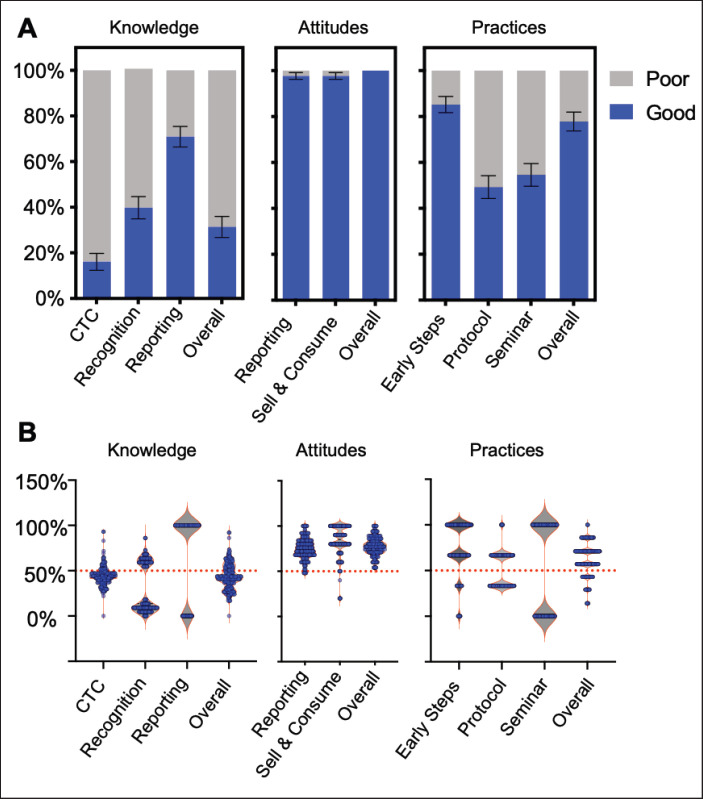
Levels of participants’ KAP on concepts about ASF. (A) The overall percentage of participants with good (blue bar) and poor (gray bar) levels of KAP subcomponents and the total of all subcomponents. Note: CTC = knowledge of ASF causative agent, transmission, and clinical signs; Error bars are 95% CI. (B) Violin plot of individual levels of KAP subcomponents and the total of all subcomponents. Note: Blue dots represent the individual participant’s KAP score in percentage; the 50% dotted line is the cutoff value used to categorize “poor” and “good” KAP scores.

The assessment of ASF CTC revealed that nearly all stakeholders were familiar with ASF (99.49%). A majority provided responses indicating partial knowledge regarding the potential causative agent (73.98%). Regarding transmission routes, a significant portion provided biomedically inaccurate responses (65.82%), such as airborne transmission, or expressed a need for further understanding by expressing no idea of how it is transmitted (3.83%). Nevertheless, some stakeholders demonstrated awareness of ASF transmission through pig meat/live pigs (33.42%), food waste (13.30%), and fomites (1.53%), with fewer mentioning ticks (1.53%) and pig feed (0.77%). Concerning clinical signs, most stakeholders recognized ASF presentation through high fever (66.33%), anorexia (54.59%), skin rashes (38.27%), and lethargy (31.63%). Additional signs, such as reddening of ears and flank (16.33%), labored breathing, and coughing (3.57%), as well as sudden death (1.02%), were also identified by some stakeholders, while a minority mentioned diarrhea, nosebleeds, vomiting, and nasal secretions. However, 2.30% of stakeholders demonstrated no knowledge of ASF clinical signs or indicated that they do not know any clinical signs of ASF. Overall, the *K* score assessment on ASF CTC indicated that most participants exhibited a poor *K* score (83.93%), while the remaining had a good score (16.07%) (Fig. 2).

Regarding ASF recognition, only 1.02 % of the stake-holders can identify all combinations of clinical signs, representing a syndromic identification of ASF. However, most participants (96.68%) can identify at least 1 of the 11 clinical signs considered in this study (see Supplementary Information 3 for KAP scoring). We also assessed if participants could identify meat potentially coming from ASF-infected pigs, of which 39.80% were able to indicate bruising or hemorrhages all over the pig meat as indicators of potential ASF infection. Overall, 60.20% of participants displayed a poor K score for ASF recognition, whereas 39.80% exhibited a good K score (Fig. 2).

Regarding reporting procedures, roughly half of the participants (57.40%) possessed accurate knowledge regarding reporting to the Municipal Agriculture Office. In comparison, 8.42% acknowledged the necessity to report suspected ASF cases to barangay officers, 4.34% indicated reporting to veterinarians, and 29.85% demonstrated no knowledge of proper reporting procedures for potential ASF cases. For ASF reporting, 70.92% of participants achieved a good K score, while 29.08% attained a poor K score (Fig. 2).

### Factors influencing the ASF knowledge score

We analyzed factors potentially influencing knowledge regarding (1) ASF CTC, (2) ASF case recognition, and (3) ASF reporting protocols. For ASF CTC, a multivariable logistic regression analysis showed a significant association of ASF CTC knowledge with participants’ age and attendance at ASF-related seminars/training, with the sex variable incorporated into the model (Table 1). Younger stakeholders (23–37 years old) were more likely to possess comprehensive knowledge concerning ASF CTC than their older counterparts (58–86 years old)) (AOR = 2.140; 95% CI = 1.012–4.526; *p* = 0.046). Furthermore, ASF-related seminar/training attendance was a significant predictor of enhanced knowledge of ASF CTC (AOR = 1.918; 95% CI = 1.038–3.546; *p* = 0.038).

**Table 1.** Logistic regression analysis of factors associated with stakeholders’ (*n* = 392) knowledge of ASF CTC in Abuyog, Leyte.

Variable	Univariate LRA			Multivariable LRA		
	OR	95% CI	*p*-value	Adjusted OR	95% CI	*p*-value
Age categories^$^						
58–86 years	Referent	–	–	Referent	–	–
49–57 years	0.723	0.304–1.722	0.463	0.612	0.245–1.531	0.294
38–48 years	0.561	0.231–1.363	0.202	0.532	0.213–1.329	0.177
23–37 years	2.440	1.201–4.959	0.014	2.140	1.012–4.526	0.046
Sex						
Male	Referent	–	–	Referent	–	–
Female	0.664	0.384–1.141	0.138	0.758	0.423–1.358	0.352
Civil Status						
Single	Referent	–	–			
Married	0.492	0.255–0.948	0.034			
Separated	0.490	0.174–1.377	0.176			
Widowed	NA	NA	NA			
Education						
Elementary	Referent	–	–			
Highschool	1.066	0.551–2.063	0.850			
Vocational	1.807	0.454–7.186	0.401			
College	2.865	1.303–6.297	0.009			
Role in the swine sector						
Farmers	Referent	–	–			
Non-farmers^#^	2.264	1.180–4.344	0.014			
Experience (years)	0.962	0.928–0.997	0.032			
Attendance at seminars or training						
Did not attend	Referent	–	–	Referent	–	–
Attended	2.367	1.315–4.260	0.004	1.918	1.038–3.546	0.038
Estimated income (Php)	1.000	1.000–1.000	0.002	1.000	1.000–1.000	0.006
Main income source						
Piggery	Referent	–	–			
Agribusiness	0.432	0.212–0.884	0.022			
*Not pig-related	0.992	0.491–2.004	0.982			

LRA = Logistic Regression Analysis; OR = Odds Ratio.

* = government jobs, sari-sari store, tuba seller, significant other’s income, pension, etc.

# = Butcher, Pig/Meat Seller, and LGU

$ = Age categories are based on percentile

NA = Not applicable/cannot be calculated due to lack of variation; Cox & Snell *R*^2^ for the multivariable LRA = 0.087.

The same analysis on knowledge of ASF case recognition showed that the participant’s role in the swine sector (i.e., farmer vs. non-farmer), participation in ASF-related seminars/training, and knowledge of ASF CTC were significant predictors, with age and sex variables included in the model (Table 2). Individuals who worked as butchers, pig/meat sellers, and LGU personnel, collectively under the non-farmer category, demonstrated a higher likelihood of possessing in-depth knowledge regarding ASF case recognition compared to farmers (AOR = 3.753; 95% CI = 1.901–7.410; *p* < 0.001). Additionally, attendance at ASF-related seminars/training emerged as a significant predictor, suggesting that individuals engaging in such educational events exhibited a better grasp of ASF case recognition (AOR = 1.869; 95% CI = 1.183–2.953; *p* = 0.007). Furthermore, participants with good basic knowledge of ASF CTC showed a significantly better knowledge of ASF case recognition (AOR = 3.254; 95% CI = CI = 1.763–6.008; *p* < 0.001).

**Table 2.** Logistic regression analysis of factors associated with stakeholders’ (*n* = 392) knowledge of ASF case recognition in Abuyog, Leyte.

Variable	Univariate LRA			Multivariable LRA		
	OR	95% CI	*p*-value	Adjusted OR	95% CI	*p*-value
Age categories^$^						
58–86 years	Referent	–	–	Referent	–	–
49–57 years	1.038	0.571–1.887	0.904	0.860	0.451–1.642	0.648
38–48 years	1.206	0.679–2.143	0.523	1.058	0.571–1.960	0.857
23–37 years	1.621	0.916–2.870	0.097	0.907	0.478–1.720	0.766
Sex						
Male	Referent	–	–	Referent	–	–
Female	0.619	0.412–0.930	0.021	0.869	0.550–1.372	0.547
Civil status						
Single	Referent	–	–			
Married	0.655	0.378–1.133	0.130			
Separated	0.463	0.204–1.050	0.065			
Widowed	NA	NA	NA			
Education						
Elementary	Referent	–	–			
Highschool	1.012	0.638–1.601	0.963			
Vocational	0.856	0.271–2.710	0.792			
College	1.182	0.615–2.272	0.616			
Role in the swine sector						
Farmers	Referent	–	–	Referent	–	–
Non-farmers^#^	5.232	2.823–9.699	<0.001	3.753	1.901–7.410	< 0.001
Experience (years)	1.003	0.979–1.027	0.816			
Attendance at seminars or training						
Did not attend	Referent	–	–	Referent	–	–
Attended	2.633	1.722–4.024	< 0.001	1.869	1.183–2.953	0.007
ASF CTC Knowledge	3.772	2.131–6.676	< 0.001	3.254	1.763–6.008	< 0.001
Estimated income (Php)	1.000	1.000–1.000	0.001			
Main income source						
Piggery	Referent	–	–			
Agribusiness	0.314	0.180–0.546	< 0.001			
*Not pig-related	0.336	0.183–0.611	< 0.001			

LRA = Logistic Regression Analysis; OR = Odds Ratio; ASF CTC = Knowledge on African swine fever causative agent, transmission, and clinical signs.

* = government jobs, sari-sari store, tuba seller, significant other’s income, pension, etc.

# = Butcher, Pig/Meat Seller, and LGU

$ = Age categories are based on percentile

NA = Not applicable/ cannot be calculated due to lack of variation; Cox & Snell *R*^2^ for the multivariable LRA = 0.136.

On knowledge of reporting ASF cases, multivariable logistic regression analysis showed a significant association of ASF reporting knowledge with attendance at ASF-related seminars/training and knowledge of ASF case recognition, with age and sex variables included in the model (Table 3). Individuals who participated in ASFrelated seminars/training showed a more comprehensive understanding of ASF case reporting (AOR = 4.391; 95% CI = 2.679–7.197; *p* < 0.001). Good knowledge of ASF case recognition was also significantly associated with good knowledge of ASF case reporting (AOR = 2.425; 95% CI = 1.427–4.122; *p* = 0.001).

Lastly, we examined which factors significantly influenced the overall knowledge of participants on ASF, as shown in Table 4. In this analysis, the overall knowledge of ASF was associated with the role of participants in the swine sector, attendance at seminars/training related to ASF, and the estimated income, with age and sex variables included in the model. Here, a collective group of non-farmers who are part of the swine sector, namely butchers, pig/meat sellers, and LGU personnel, showed a better overall knowledge of ASF than pig farmers (AOR = 2.260; 95% CI = 1.163–4.391; *p* = 0.016). Attendance at ASF-related seminars/ training positively influenced overall knowledge of ASF (AOR = 3.629; 95% CI = 2.168–6.076; *p* < 0.001). While estimated income may appear significantly associated with the overall knowledge, it showed no impact on the direction of the knowledge score (AOR = 1.000; 95% CI = 1.000–1.000; *p* = 0.048).

**Table 3.** Logistic regression analysis of factors associated with stakeholders’ (*n* = 392) knowledge of ASF case reporting in Abuyog, Leyte.

Variable	Univariate LRA			Multivariable LRA		
	OR	95% CI	*p*-value	Adjusted OR	95% CI	*p*-value
Age categories^$^						
58–86 years	Referent	–	–	Referent	–	–
49–57 years	1.185	0.639–2.200	0.590	1.030	0.525–2.022	0.931
38–48 years	1.161	0.639–2.111	0.624	1.058	0.553–2.026	0.864
23–37 years	1.625	0.871–3.033	0.127	1.162	0.585–2.308	0.669
Sex						
Male	Referent	–	–	Referent	–	–
Female	0.711	0.456–1.107	0.131	0.880	0.540–1.437	0.610
Civil Status						
Single	Referent	–	–			
Married	0.715	0.380–1.345	0.298			
Separated	0.781	0.322–1.894	0.585			
Widowed	NA	NA	NA			
Education						
Elementary	Referent	–	–			
Highschool	0.998	0.614–1.621	0.994			
Vocational	1.131	0.333–3.835	0.843			
College	2.212	0.981–4.986	0.056			
Role in the swine sector						
Farmers	Referent	–	–			
Non-farmers^#^	1.379	0.725–2.627	0.327			
Experience (years)	0.986	0.961–1.012	0.302			
Attendance at seminars or training						
Did not attend	Referent	–	–	Referent	–	–
Attended	5.181	3.157–8.504	<0.001	4.391	2.679–7.197	<0.001
ASF CTC Knowledge	2.812	1.339–5.911	0.006			
ASF Recognition Knowledge	3.172	1.920–5.242	<0.001	2.425	1.427–4.122	0.001
Estimated income (Php)	1,000	1.000–1.000	0.015			
Main income source						
Piggery	Referent	–	–			
Agribusiness	–	0.462	0.244–0.873	0.017		
*Not pig-related	0.807	0.399–1.631	0.551			

LRA = Logistic Regression Analysis; OR = Odds Ratio; ASF CTC = African swine fever causative agent, transmission, and clinical signs.

* = government jobs, sari-sari store, tuba seller, significant other’s income, pension, etc.

# = Butcher, Pig/Meat Seller, and LGU

$ = Age categories are based on percentile

NA = Not applicable/ cannot be calculated due to lack of variation; Cox & Snell *R*^2^ for the multivariable LRA = 0.146.

**Table 4.** Logistic regression analysis of factors associated with stakeholders’ (*n* = 392) overall knowledge of ASF in Abuyog, Leyte.

Variable	Univariate LRA			Multivariable LRA		
	OR	95% CI	*p*-value	Adjusted OR	95% CI	*p*-value
Age categories^$^						
58–86 years	Referent	–	–	Referent	–	–
49–57 years	0.774	0.404–1.485	0.441	0.567	0.277–1.159	0.120
38–48 years	0.998	0.541–1.840	0.995	0.816	0.418–1.595	0.552
23–37 years	1.770	0.981–3.193	0.058	1.173	0.606–2.272	0.635
Sex						
Male	Referent	–	–	Referent	–	–
Female	0.616	0.401–0.947	0.027	0.841	0.513–1.378	0.493
Civil Status						
Single	Referent	–	–			
Married	0.502	0.287–0.879	0.016			
Separated	0.500	0.217–1.151	0.103			
Widowed	NA	NA	NA			
Education						
Elementary	Referent	–	–			
Highschool	1.200	0.732–1.968	0.469			
Vocational	1.035	0.304–3.525	0.956			
College	1.698	0.862–3.346	0.126			
Role in the swine sector						
Farmers	Referent	–	–	Referent	–	–
Non-farmers^#^	4.426	2.484–7.884	< 0.001	2.260	1.163–4.391	0.016
Experience (years)	0.990	0.964–1.016	0.433			
Attendance at seminars or training						
Did not attend	Referent	–	–	Referent	–	–
Attended	4.550	2.787–7.428	<0.001	3.629	2.168–6.076	<0.001
Estimated income (Php)	1.000	1.000–1.000	0.002	1.000	1.000–1.000	0.048
Main income source						
Piggery	Referent	–	–			
Agribusiness	0.244	0.139–0.431	<0.001			
*Not pig-related	0.404	0.223–0.733	0.003			

LRA = Logistic Regression Analysis; OR = Odds Ratio; ASF CTC = African swine fever causative agent, transmission, and clinical signs.

* = government jobs, sari-sari store, tuba seller, significant other’s income, pension, etc.

# = Butcher, Pig/Meat Seller, and LGU

$ = Age categories are based on percentile

NA = Not applicable/ cannot be calculated due to lack of variation; Cox & Snell *R*^2^ for the multivariable LRA = 0.150.

### ASF attitudes score

Attitudes encompass stakeholders’ tendency to report suspected ASF cases and their sentiments regarding selling and consuming potentially infected pigs or meat. The latter serves as an indirect measure of non-reporting, given that stakeholders may opt to dispose of pigs and meat exhibiting signs indicative of ASF.

The assessment of attitudes toward reporting revealed that most stakeholders were likely to report possible ASF cases to the appropriate authorities (48.72% agree; 40.82% Strongly agree). However, attitudes toward case reporting were polarized, with nearly half expressing a preference against reporting. Indeed, a significant portion of stakeholders needed confirmation that clinical signs indicated ASF before contacting the proper authorities (51.79% agree; 5.36% strongly agree). Moreover, a considerable percentage (42.09% agree; 37.76% strongly agree) recognized their dependency on proper authorities for handling ASF cases. The majority of participants agreed (43.11%) that reporting cases would adversely affect their income sources. Overall, stakeholders demonstrated good attitude scores in ASF reporting (Fig. 2), indicating their readiness to report suspected ASF cases through appropriate channels.

The stakeholders’ attitudes toward selling and consuming live pigs and meat from suspected ASF-infected pigs were also evaluated. Most participants disagreed with consuming meat from potentially infected pigs (39.13% disagree; 35.04% strongly disagree), and a subset indicated agreement with consuming infected meat (14.58% agree; 1.53% strongly agree). Additionally, a significant percentage disagreed with selling potentially infected products (37.24% disagree; 58.42% strongly disagree). Overall, participants exhibited good attitude scores (Fig. 2) indicative of an aversion to selling and consuming infected pigs and meat, with the vast majority expressing a preference against engaging in these activities.

### Factors influencing ASF attitude score

A multivariable logistic regression analysis was only performed on attitudes toward reporting ASF cases, as other attitude scores showed no variations in scores or dominantly showed a “good” category. Here, ASF case reporting attitude was associated with the overall knowledge of ASF, with age and sex variables included in the model (Table 5). A good overall ASF knowledge score was significantly associated with a good attitude in terms of ASF case reporting (AOR = 5.706; 95% CI = 2.059–15.815; *p* < 0.001).

**Table 5.** Logistic regression analysis of factors associated with stakeholders’ (*n* = 392) attitudes toward ASF case reporting in Abuyog, Leyte.

Variable	Univariate LRA			Multivariable LRA		
	OR	95% CI	*p*-value	Adjusted OR	95% CI	*p*-value
Age categories^$^						
58–86 years	Referent	–	–	Referent	–	–
49–57 years	NA	NA	NA	NA	NA	NA
38–48 years	1.075	0.212–5.461	0.930	1.264	0.218–7.332	0.794
23–37 years	1.065	0.210–5.407	0.940	0.754	0.132–4.295	0.751
Sex						
Male	Referent	–	–	Referent	–	–
Female	0.336	0.069–1.638	0.177	0.513	0.094–2.806	0.441
Civil Status						
Single	Referent	–	–			
Married	0.643	0.078–5.320	0.682			
Separated	0.661	0.040–10.872	0.772			
Widowed	NA	NA	NA			
Education						
Elementary	Referent	–	–			
Highschool	0.270	0.032–2.270	0.228			
Vocational	0.107	0.006–1.821	0.122			
College	0.430	0.026–7.002	0.553			
Role in the swine sector						
Farmers	Referent	–	–			
Non-farmers^#^	NA	NA	NA			
Experience (years)	1.029	0.944–1.122	0.519			
ASF Knowledge Score	6.11	2.223–16.830	< 0.001	5.706	2.059–15.815	<0.001
Estimated income (Php)	1.000	1.000–1.000	0.153			
Main income source						
Piggery	Referent	–	–			
Agribusiness	0.754	0.153–3.715	0.729			
*Not pig-related	NA	NA	NA			

LRA = Logistic Regression Analysis; OR = Odds Ratio.

* = government jobs, sari-sari store, tuba seller, significant other’s income, pension, etc.

# = Butcher, Pig/Meat Seller, and LGU

$ = Age categories are based on percentile; NA = Not applicable/ cannot be calculated due to lack of variation of the categorized dependent variable (e.g., All responses are categorized as ‘good’); Cox & Snell *R*^2^ for the multivariable LRA = 0.057.

### ASF practice score

The assessment of practices related to early steps of case reporting revealed a good practice score among participants (85.20%) (Fig. 2). The majority of participants (76.02%) acknowledged seeking consultation with a veterinarian when pigs presented clinical signs. Concerning the timeline for notifying authorities, 77.30% of respondents reported informing authorities within less than 7 days, which still qualifies for early reporting [19]. However, 22.70% of participants answered a delayed notification beyond 7 days. Additionally, 74.49% admitted to slaughtering pigs that display potential clinical signs of ASF.

Regarding the protocol for case reporting, 9.69% answered that suspected ASF cases could be reported to the Municipal Agriculture Office and 6.63% to barangay officers, but the majority (83.67%) may have opted out of reporting potential ASF cases. Stakeholders who observed ASF cases but refrained from reporting cited reasons such as avoiding conflict with neighbors (36.48%), while 63.52% chose not to report without specifying motives. Overall, almost half of the participants (49.23%) showed good practice in the ASF reporting protocol (Fig. 2).

We also determined knowledge-seeking practices through attendance in ASF-related seminars or training. A substantial number of participants (45.41%) indicated no attendance in seminars or training sessions related to ASF. Our results also showed that 51.65% of pig farmers indicated no prior seminars or training regarding ASF, in contrast to only 10.17% of non-farmer participants. As to the conduct of seminars or training, 11.48% participated through the Municipal Agriculture Office, 32.14% through barangay officers, and 10.97% through other means, including seminars conducted by the National Meat Inspection Service and the DA-Babay ASF training program. Overall, by covering practices on early steps of reporting, protocol of reporting, and knowledge-seeking practices, 77.04% of participants exhibited a good ASF practice score. In comparison, 22.96% demonstrated a poor ASF practice score (Fig. 2).

### Factors influencing ASF practice score

We analyzed factors potentially influencing practices in the early steps of case reporting, the protocol of ASF reporting, and knowledge-seeking practices through ASF-related seminars/training. For the early steps of case reporting, a multivariable logistic regression analysis showed a significant association with the role of participants in the swine sector and overall attitude score, with age, sex, and overall knowledge score variables included in the model (Table 6). Participants who worked as butchers, pig/meat sellers, and LGU personnel, collectively under the non-farmer category, demonstrated better case reporting practices compared to farmers (AOR = 5.244; 95% CI = 1.179–23.328; *p* = 0.030). However, our analysis showed that participants’ attitude toward reporting cases was inversely associated with the actual practice of early steps in case reporting (AOR = 0.846; 95% CI = 0.771–0.927; *p* < 0.001).

**Table 6.** Logistic regression analysis of factors associated with stakeholders’ (*n* = 392) practices on early steps of ASF reporting in Abuyog, Leyte.

Variable	Univariate LRA			Multivariable LRA		
	OR	95% CI	p-value	Adjusted OR	95% CI	p-value
Age categories^$^						
58–86 years	Referent	–	–	Referent	–	–
49–57 years	1.183	0.546–2.566	0.670	1.279	0.569–2.872	0.551
38–48 years	2.000	0.867–4.620	0.104	1.915	0.803–4.567	0.143
23–37 years	1.076	0.514–2.252	0.846	1.130	0.510–2.501	0.764
Sex						
Male	Referent	–	–	Referent	–	–
Female	1.242	0.711–2.170	0.447	1.437	0.787	2.626
Civil Status						
Single	Referent	–	–			
Married	0.968	0.445–2.108	0.935			
Separated	0.833	0.284–2.443	0.740			
Widowed	NA	NA	NA			
Education						
Elementary	Referent	–	–			
Highschool	1.368	0.715–2.618	0.344			
Vocational	0.332	0.100–1.100	0.071			
College	0.793	0.341–1.845	0.591			
Role in the swine sector						
Farmers	Referent	–	–	Referent	–	–
Non-farmers#	5.762	1.367–24.293	0.017	5.244	1.179–23.328	0.030
Experience (years)	0.998	0.966–1.032	0.924			
ASF knowledge score	1.224	0.906–1.654	0.187	1.390	0.977–1.978	0.067
ASF attitudes score	0.866	0.798–0.941	<0.001	0.846	0.771–0.927	<0.001
Estimated income (Php)	1.000	1.000–1.000	0.255			
Main income source						
Piggery	Referent	–	–			
Agribusiness	0.388	0.145–1.040	0.060			
*Not pig-related	0.326	0.118–0.903	0.031			

LRA = Logistic Regression Analysis; OR = Odds Ratio.

* = government jobs, sari-sari store, tuba seller, significant other’s income, pension, etc.

# = Butcher, Pig/Meat Seller, and LGU; NA = Not applicable/ cannot be calculated due to lack of variation of the categorized dependent variable

$ = Age categories are based on percentile; Cox & Snell *R*2 for the multivariable LRA = 0.073.

In the ASF reporting protocol, a multivariable logistic regression analysis showed a significant association with the overall ASF knowledge score, with age and sex variables included in the model (Table 7). Good practice in ASF case reporting was significantly associated with enhanced overall knowledge of ASF (AOR = 1.648; 95% CI = 1.308–2.073; *p* < 0.001). The same analysis conducted on knowledge-seeking practices by attending seminars and training showed a significant association with participants’ attitudes toward ASF reporting and their primary source of income, as adjusted with age and sex variables in the model (Table 8). A good attitude toward ASF reporting translated into good knowledge-seeking practice by attending ASF-related seminars/training (AOR = 1.102; 95% CI = 1.034–1.174; *p* = 0.003). Participants whose main source of livelihood is not pig farming were likely to have poor knowledge-seeking practices (i.e., agribusiness but not piggery: AOR = 0.062; 95% CI = 0.025–0.153; *p* < 0.001; not pig-related main income source: AOR = 0.100; 95% CI = 0.039–0.253; *p* < 0.001).

**Table 7.** Logistic regression analysis of factors associated with stakeholders’ (*n* = 392) practices on the protocol of ASF reporting in Abuyog, Leyte.

Variable	Univariate LRA			Multivariable LRA		
	OR	95% CI	*p*-value	Adjusted OR	95% CI	*p*-value
Age categories^$^						
58–86 years	Referent	–	–	Referent	–	–
49–57 years	1.435	0.807–2.553	0.219	1.459	0.806–2.640	0.212
38–48 years	1.167	0.667–2.040	0.589	1.167	0.656–2.076	0.598
23–37 years	1.446	0.826–2.533	0.197	1.274	0.707–2.296	0.420
Sex						
Male	Referent	–	–	Referent	–	–
Female	1.027	0.690–1.528	0.897	1.219	0.801–1.857	0.355
Civil Status						
Single	Referent	–	–			
Married	0.757	0.437–1.309	0.319			
Separated	0.600	0.273–1.319	0.204			
Widowed	NA	NA	NA			
Education						
Elementary	Referent	–	–			
Highschool	1.302	0.829–2.046	0.252			
Vocational	1.302	0.430–3.939	0.640			
College	1.834	0.955–3.525	0.069			
Role in the swine sector						
Farmers	Referent	–	–			
Non-farmers^#^	0.783	0.449–1.367	0.390			
Experience (years)	1.000	0.976–1.023	0.970			
ASF knowledge score	1.631	1.303–2.043	< 0.001	1.648	1.308–2.073	<0.001
ASF attitudes score	1.025	0.970–1.083	0.375			
Estimated income (Php)	1.000	1.000–1.000	0.403			
Main income source						
Piggery	Referent	–	–			
Agribusiness	1.059	0.620–1.809	0.833			
*Not pig-related	1.171	0.656–2.089	0.593			

LRA = Logistic Regression Analysis; OR = Odds Ratio.

* = government jobs, sari-sari store, tuba seller, significant other’s income, pension, etc.

# = Butcher, Pig/Meat Seller, and LGU; NA = Not applicable/ cannot be calculated due to lack of variation of the categorized dependent variable

$ = Age categories are based on percentile; Cox & Snell *R*^2^ for the multivariable LRA = 0.054.

**Table 8.** Logistic regression analysis of factors associated with stakeholders’ (*n* = 392) knowledge-seeking practices through attendance in seminars and training related to ASF in Abuyog, Leyte.

Variable	Univariate LRA			Multivariable LRA		
	OR	95% CI	*p*-value	Adjusted OR	95% CI	*p*-value
Age categories^$^						
58–86 years	Referent	–	–	Referent	–	–
49–57 years	1.507	0.846–2.862	0.163	1.052	0.562–1.970	0.874
38–48 years	1.253	0.718–2.187	0.428	0.854	0.462–1.581	0.616
23–37 years	2.167	1.224–3.835	0.008	1.160	0.612–2.201	0.648
Sex						
Male	Referent	–	–	Referent	–	–
Female	0.636	0.435–0.952	0.028	0.758	0.478–1.203	0.240
Civil Status						
Single	Referent	–	–			
Married	0.513	0.289–0.915	0.024			
Separated	0.465	0.208–1.040	0.062			
Widowed	NA	NA	NA			
Education						
Elementary	Referent	–	–			
Highschool	1.151	0.733–1.806	0.542			
Vocational	1.631	0.517–5.149	0.404			
College	0.941	0.494–1.794	0.854			
Role in the swine sector						
Farmers	Referent	–	–			
Non-farmers^#^	9.437	3.949–22.550	< 0.001			
Experience (years)	0.994	0.970–1.018	0.605			
ASF attitudes score	1.112	1.050–1.178	< 0.001	1.102	1.034–1.174	0.003
Estimated income (Php)	1.000	1.000–1.000	0.032			
Main income source						
Piggery	Referent	–	–	Referent	–	–
Agribusiness	0.062	0.026–0.149	< 0.001	0.062	0.025–0.153	< 0.001
*Not pig-related	0.102	0.041–0.254	< 0.001	0.100	0.039–0.253	< 0.001

LRA = Logistic Regression Analysis; OR = Odds Ratio.

* = government jobs, sari-sari store, tuba seller, significant other’s income, pension, etc.

# = Butcher, Pig/Meat Seller, and LGU; NA = Not applicable/ cannot be calculated due to lack of variation of the categorized dependent variable

$ = Age categories are based on percentile; Cox and Snell *R*^2^ for the multivariable LRA = 0.183.

Lastly, we analyzed the overall ASF practices, encompassing reporting and knowledge-seeking behavior, as shown in Table 9. In this analysis, the overall practices were associated with the overall knowledge and attitude scores, as well as where participants derived their primary source of income, with age and sex variables included in the model. Here, a good overall knowledge score influenced good practice on case reporting (AOR = 2.683; 95% CI = 1.912–3.765; *p* < 0.001). However, attitudes toward case reporting showed an inverse association with the actual case reporting practices (AOR = 0.883; 95% CI = 0.814–0.957; *p* = 0.002). In addition, participants whose main livelihood is not pig farming were likely to have poor practices on ASF case reporting (i.e., not pig-related main income source: AOR = 0.282; 95% CI = 0.107–0.746; *p* = 0.011).

**Table 9.** Logistic regression analysis (LRA) of factors associated with stakeholders’ (*n* = 392) overall practices toward ASF case reporting in Abuyog, Leyte.

Variable	Univariate LRA			Multivariable LRA		
	OR	95% CI	*p*-value	Adjusted OR	95% CI	*p*-value
Age categories^$^						
58–86 years	Referent	–	–	Referent	–	–
49–57 years	1.174	0.602–2.288	0.638	1.214	0.588–2.505	0.600
38–48 years	1.663	0.840–3.292	0.144	1.726	0.812–3.666	0.155
23–37 years	1.209	0.631–2.318	0.567	1.186	0.571–2.465	0.647
Sex						
Male	Referent	–	–	Referent	–	–
Female	0.970	0.601–1.567	0.902	1.334	0.771–2.310	0.303
Civil Status						
Single	Referent	–	–			
Married	0.832	0.418–1.658	0.602			
Separated	0.588	0.235–1.473	0.258			
Widowed	NA	NA	NA			
Education						
Elementary	Referent	–	–			
Highschool	1.519	0.884–2.611	0.130			
Vocational	0.613	0.191–1.969	0.411			
College	0.949	0.455–1.977	0.889			
Role in the swine sector						
Farmers	Referent	–	–			
Non-farmers^#^	2.349	1.026–5.377	0.043			
Experience (years)	0.996	0.968–1.025	0.783			
ASF knowledge score	2.294	1.695–3.106	<0.001	2.683	1.912–3.765	<0.001
ASF attitudes score	0.948	0.886–1.013	0.116	0.883	0.814–0.957	0.002
Estimated income (Php)	1.000	1.000–1.000	0.411			
Main income source						
Piggery	Referent	–	–	Referent	–	–
Agribusiness	0.282	0.115–0.690	0.006	0.447	0.175–1.146	0.094
*Not pig-related	0.226	0.090–0.569	0.002	0.282	0.107–0.746	0.011

LRA = Logistic Regression Analysis; OR = Odds Ratio; ASF CTC = African swine fever causative agent, transmission, and clinical signs.

* = government jobs, sari-sari store, tuba seller, significant other’s income, pension, etc.

# = Butcher, Pig/Meat Seller, and LGU; NA = Not applicable/ cannot be calculated

$ = Age categories are based on percentile; Cox and Snell *R*^2^ for the multivariable LRA = 0.135.

## Discussion

The study determines stakeholders’ KAPs regarding ASF in Abuyog, Leyte, Philippines, the site of the first ASF case in the Visayas. Prior experience with ASF indicates that KAPs of stakeholders regarding case reporting are pivotal factors in early case detection, thereby facilitating timely interventions to contain outbreaks and mitigate the geographical spread of the virus [15,20,21].

Our findings indicate that most stakeholders had poor knowledge regarding concepts surrounding the CTC of ASF, as well as disease recognition. However, they have good knowledge regarding ASF reporting. This finding is similar to the recent KAP study in the neighboring administrative area, showing that participants lacked knowledge about ASF CTC and disease recognition but possessed sufficient knowledge about ASF reporting [9]. The result indicates a concentrated government effort on the ASF reporting protocol but a lesser focus on the crucial aspect of disease recognition, which forms the foundation for reporting a case. Muñoz-Gómez et al. [5] observed that backyard farmers may struggle to comprehend ASF concepts, indicating the necessity for improved information dissemination to these stakeholders. Hence, ASF Information, Education, and Communication (IEC) campaigns need continual reassessment to integrate innovative approaches for educating farmers [22]. One of the successful stories of IECs was in Baybay City, when extensive campaigns against swill feeding may have contributed to a six-fold reduction in its practice [9,23].

Beyond levels of knowledge, we have identified several factors that may account for differences in participants’ understanding of ASF. In Abuyog, attending ASF seminars or training is strongly linked to an improved understanding of ASF CTC, disease recognition, and case reporting, which in turn enhances overall ASF knowledge. Participants who participated in ASF-related seminars and training exhibited a greater ability to recognize the clinical presentation of ASF, significantly strengthening their knowledge about case reporting. Thus, in Abuyog, overall knowledge of ASF is associated with good case reporting practice. Our analysis suggests that knowledge-seeking behavior is associated with participants whose primary source of income is pig farming, plus a favorable attitude toward ASF reporting. However, it is crucial to acknowledge that many pig farmers indicate an absence of prior seminars or trainings related to ASF, suggesting either that they may lack access to these activities or the motivation to participate.

Additionally, we noted an association between higher knowledge of ASF CTC and younger stakeholders, which is expected, as younger individuals tend to be more adept at using computers and smartphones [24]. Conversely, the ability to recognize ASF is significantly affected by the type of stakeholder, with farmers generally exhibiting lower knowledge of identifying ASF cases on their farms. Our findings indicate that enhancing understanding of basic ASF concepts could help improve knowledge of disease recognition, and such improvement can be achieved through seminars and training sessions. However, the challenge lies in designing these seminars and training programs to boost attendance and engagement among pig farmers and key players in pig production. Na et al. [25] reported that pig farmers rely heavily on knowledge gained through production experience and usually do not proactively plan for unexpected challenges. Hence, knowledge gaps persist even with various training sessions and workshops to raise awareness [5]. This underscores the need for more effective educational strategies and proactive planning measures to better equip stakeholders in managing ASF.

Although most participants exhibit positive attitudes toward reporting ASF cases, many prefer confirmation that the clinical presentation suggests ASF before reporting to authorities. In this context, a “confirmation” may mean a condition by which farmers observe that they experience a similar clinical presentation of the outbreak with neighboring pig farms, creating a consensus among farmers to report when they feel unable to manage the outbreaks on their own. This cautious stance is primarily shaped by the significant financial repercussions of ASF and tensions with neighboring farms resulting from the depopulation of both infected and adjacent non-infected farms [8, 26]. Additionally, there may also be political pressure to underreport cases to avoid economic consequences at the LGU level, as transports of pigs, pork, and related products can be halted through a zoning system imposed by the national government [19]. Of note, farmers’ attitudes toward ASF reporting are generally more positive, driven by the direct and immediate impact of ASF on their livelihoods.

While practices on case reporting appear encouraging for the majority of participants, many also show reservations about reporting to authorities after observing suspected ASF cases. In the early stage of ASF outbreaks, our data suggest that pig farmers are less likely to report than other stakeholders and that good attitudes may not translate into a case reporting practice. An analysis of the protocol indicates that good overall knowledge translates into good practices, not good attitudes. Another layer of analysis also suggests that overall practices are influenced by the primary source of income, indicating that those participants whose main livelihoods are not pig farming are likely to have poor ASF-related practices. This suggests that the financial impact of ASF on stakeholders is a significant factor in the actual reporting of cases.

Non-reporting could be attributed to fear of pig depopulation measures, including pigs not showing clinical signs, being enforced within a specific radius of confirmed cases, as per the DA Memorandum Order No. 22, series 2020 [27]. Local leaders and pig farmers may argue that this policy is detrimental to the local and national economy and is prejudicial against the poor. Similarly, pig farmers in Estonia have expressed similar sentiments [28]. Stakeholders emphasize that the depopulation policy is causing significant mental stress for affected farmers and animal health workers [26]. Additionally, the adequacy and timeliness of financial compensation may have influenced the willingness to report ASF. Effective compensation policies are crucial for prevention and early reporting and require thorough socioeconomic analysis. Without this, payment policies may either incentivize tolerating outbreaks or discourage reporting due to fear of economic loss. Often, compensation policies are narrower, failing to account for the diverse socioeconomic contexts within the pork value chain [29].

It is important to note that the data come from stakeholders’ responses, reflecting their subjective views and opinions. These views may change and vary depending on geographical locations. However, one of the significant advantages of this KAP study is its ability to capture key stakeholders’ basic KAPs. This understanding is crucial for developing tailored ASF-related programs. Using the insights from the KAP results, we can effectively customize these programs to meet the specific needs and nuances of the local context, maximizing their effectiveness and impact.

## Conclusion

The majority of key stakeholders within the study site display a lack of knowledge regarding the CTC signs of ASF, potentially resulting in inadequate disease recognition. Despite this, most stakeholders strongly understand the process for reporting ASF cases and demonstrate favorable attitudes and practices toward reporting. This study has also identified potential factors influencing poor and good KAPs toward ASF concepts and case reporting. Participation in ASF seminars or training is significantly associated with a better knowledge of basic ASF concepts, disease recognition, and case reporting. Those who attended ASF-related seminars and training demonstrated an improved capacity to recognize the clinical signs of ASF, leading to enhanced knowledge about reporting ASF cases. Hence, knowledge of ASF is linked to effective case-reporting practices on the study site. However, knowledge of ASF disease recognition is generally low among pig farmers. There are significant reservations regarding the reporting of suspected ASF cases to authorities despite the overall positive attitudes. Nevertheless, ASF knowledge translates into case reporting practices, which are also driven by financial factors.

## Data Availability

The supplementary information 1 to 4 can be found by requesting the corresponding author.

## List of abbreviations

ASF, African swine fever

AOR, adjusted odds ratio

CTC, causative agent, transmission, and clinical signs

DA-LGU, Department of Agriculture-Local Government Unit

KAP, knowledge, attitudes, and practices

LGU, Local Government Unit

OR, odds ratio.

## SUPPLEMENTARY MATERIAL

The supplementary material can be accessed at the journal’s website
